# Differences between the Cell Populations from the Peritenon and the Tendon Core with Regard to Their Potential Implication in Tendon Repair

**DOI:** 10.1371/journal.pone.0092474

**Published:** 2014-03-20

**Authors:** Jennifer A. Cadby, Evelyne Buehler, Charles Godbout, P. René van Weeren, Jess G. Snedeker

**Affiliations:** 1 Department of Orthopaedics, Balgrist Hospital, University of Zurich, Zurich, Switzerland; 2 Department Health Sciences and Technology, Swiss Federal Institute of Technology, Zurich, Switzerland; 3 Department of Equine Sciences, Faculty of Veterinary Medicine, Utrecht University, Utrecht, The Netherlands; 4 Osteoarthritis Research Unit, University of Montreal Hospital Research Centre (CRCHUM), Montreal, Quebec, Canada; Scuola Superiore Sant'Anna, Italy

## Abstract

The role of intrinsic and extrinsic healing in injured tendons is still debated. In this study, we characterized cell plasticity, proliferative capacity, and migration characteristics as proxy measures of healing potential in cells derived from the peritenon (extrinsic healing) and compared these to cells from the tendon core (intrinsic healing). Both cell populations were extracted from horse superficial digital flexor tendon and characterized for tenogenic and matrix remodeling markers as well as for rates of migration and replication. Furthermore, colony-forming unit assays, multipotency assays, and real-time quantitative polymerase chain reaction analyses of markers of osteogenic and adipogenic differentiation after culture in induction media were performed. Finally, cellular capacity for differentiation towards a myofibroblastic phenotype was assessed. Our results demonstrate that both tendon- and peritenon-derived cell populations are capable of adipogenic and osteogenic differentiation, with higher expression of progenitor cell markers in peritenon cells. Cells from the peritenon also migrated faster, replicate more quickly, and show higher differentiation potential toward a myofibroblastic phenotype when compared to cells from the tendon core. Based on these data, we suggest that cells from the peritenon have substantial potential to influence tendon-healing outcome, warranting further scrutiny of their role.

## Introduction

Injuries to energy-storing tendons are prevalent in athletes as well as in the general population. It has been estimated that tendinopathy accounts for 30% to 50% of all injuries related to sports [1]. The most common causes of tendon ailments are acute trauma or repetitive activities that create an accumulation of micro-injuries in the tendon tissue [2]. Tendinopathy is a result of a deficient healing response to these accumulated micro-injuries in the tendon tissues, which for largely unknown reasons are unable to effectively regenerate [3]. Although many medical options are available to treat tendon injuries, there is a high recurrence rate and the prognosis for returning to previous performance levels is still poor. A better understanding of the cellular mechanisms involved in the natural healing of tendons could enable improved medical treatment.

It was first suggested that tendons lack the capacity for intrinsic healing and that in-growth of cells from the surrounding tissues is necessary to enable healing of tendon injuries [4], [5]. The tendon is surrounded by the paratenon, a loose fibrillar tissue that functions as an elastic sleeve permitting free movement of the tendon against other tissues [6]. Under the paratenon, the entire tendon is surrounded by a fine connective tissue sheath called epitenon [6]. The paratenon and the epitenon form together the peritenon. Later work demonstrated the capacity of tendons to heal intrinsically [7]–[10], and it is now believed that both intrinsic and extrinsic healing play a synergistic role in tendon regeneration [11], [12]. However, the extent of the contribution of each is still not well defined. While intrinsic healing capacity is commonly reported as being inferior [13], it remains unknown whether this could be due to a more limited regenerative capacity of the resident cell population.

Another question that remains unanswered is whether aberrant healing is related to the nature of cells migrating towards the injured area, either from the surrounding tissue or from the tendon core. Cells with a multi-lineage differentiation potential are credited with the capacity to naturally remodel, repair, and regenerate various tissue types when necessary [14]. However, the multi-lineage differentiation potential of cells can also underlie pathological processes when differentiation is not in accordance with tissue function (ectopic differentiation) [15]. Fat deposition as well as calcification has been observed in clinical cases of tendinopathy [16], [17]. Furthermore, during extensive tissue remodeling, fibroblasts may acquire the phenotype of myofibroblasts. Briefly, myofibroblasts have stress fibers that incorporate alpha smooth muscle actin (α-SMA), which facilitates forces required for wound contraction [18]. Myofibroblasts also synthesize abundant amounts of collagen and are believed to be responsible for the formation of persistent scar tissue (fibrosis) and the shrinkage of peritendinous tissue [19], [20]

In this study, we compared the potential healing capacity of cell populations carefully isolated from the tendon core or the peritenon tissues of horse superficial digital flexor tendons (SDFT). We first investigated differences in gene expression between these two cell populations based on tenogenic markers. We then compared their migration and replication rates, as well as their capacity to produce collagen, as indicators of their healing potential. Additionally, our interest was also to assess their potential to differentiate towards osteogenic, adipogenic and myofibroblastic phenotypes, as this might relate to their potential to adversely affect healing outcome.

## Methods

### Isolation of cells from the core of the tendon and from the peritenon

All animal tissues were obtained from animals being sacrificed for food purposes and, by state (Canton of Zurich) and federal (Swiss) regulations, no ethical approval was required. SDFTs were collected from horses that had been freshly slaughtered for their meat in local abattoirs (Boucherie chevaline Estavayer-le-lac and Metzger Dürrenäsch, both in Switzerland; permissions were given from the slaughterhouses to use these animal parts). The horse SDFT has a characteristic peritenon overlying the tendon core. Cells were extracted either from the loose peritenon tissue or from the core of the tendon, leaving 2 mm of the edge in order to obtain two cell populations with clearly distinct tissue origins. Tendon cells were isolated by digestion of the tendon matrix using Protease Type XIV (Sigma-Aldrich, St. Louis, MO) for 2 h at 37°C and Collagenase B solution (Roche, Burgess Hill, UK) for 16 h at 37°C. After matrix digestion, the mixture was filtered and centrifuged at 400 g for 8 min at room temperature. The cell pellet was re-suspended and cultured at 37°C, 5% CO_2_ in expansion medium (Dulbecco's modified Eagle's medium (DMEM), 10% fetal calf serum (FCS), 50 μg/ml gentamicin and 1.5 μg/ml fungizone (all from Life technologies, Paisley, UK)), unless stated otherwise. Cells were cultured and trypsinized at subconfluency and only cells freshly digested or from the first passage were used for the experiments listed below. For *ex vivo* differentiation experiments, explants of horse SDFT (size 10×2×2 mm) with the peritenon still surrounding the tendon were used.

### Migration assay

For these experiments, cells from twelve different horses were analyzed. Using 6-well plate dishes, cells were seeded at a density of 10,000 cells/cm^2^ in each well and were maintained at 37°C and 5% CO_2_ for 6 days to permit cell adhesion and the formation of a confluent monolayer. The confluent monolayer was then scored with a sterile pipette microtip to leave a scratch of ∼0.4–0.5 mm in width. The scratch areas were monitored on an inverted Zeiss microscope (Observer Z1, Oberkochen, Germany) by collecting digitized pictures at various time points until closure was complete. The images were then analyzed using Image J software (National Institute of Health, Bethesda, MD). All scratch assays were performed in duplicate. The migration rate was calculated by dividing the newly covered area by twice the width of the lesion in order to determine the average distance the cells had covered to close the wound. This distance was then divided by time to calculate the migration speed in μm/h.

### Replication assay

Tendon and peritenon cells (passage 0) from four different horses were plated at 10,000 cells/cm^2^ in 3 flasks each. After 7 days in culture, the cells were trypsinized and counted using Trypan blue to exclude dead cells.

### Colony-forming unit assay

Tendon and peritenon cells from seven horses were plated at 50 cells/cm^2^ in Petri dishes. After nine days in culture, colonies were counted and characterized as previously described by Franken et al. [21]. Briefly, cells were rinsed with PBS, fixed in 4% paraformaldehyde, stained with 0.5% crystal violet for 30 min and rinsed twice with water. Size and number of the colonies were evaluated.

### Differentiation experiments

Eight horses were used for differentiation experiments. For tenogenic control cultures, the cells were cultivated in expansion medium with a seeding density of 10,000 cells/cm^2^. The medium was renewed every 2–3 days and the cells were kept in a 5% CO_2_ environment at 37°C. To induce differentiation, we followed protocols established by others [22]. Briefly, to induce osteogenic differentiation, the cells were seeded at a density of 3,000 cells/cm^2^ and cultured in DMEM High Glucose with Glutamax (Life technologies), 10% FCS (Life technologies), 0.6% fungizone (Life technologies), 0.1% gentamicin (Life technologies) and freshly added 10 mM glycerol phosphate (Sigma-Aldrich), 0.1 μM dexamethasone (Sigma-Aldrich) and 0.1 mM L-ascorbic acid 2-phosphate (Sigma-Aldrich). To induce adipogenic differentiation cells were seeded at 20,000 cells/cm^2^ and the induction medium consisted of DMEM Glutamax (Life technologies) with 10% FCS (Life technologies), 0.6% fungizone (Life technologies), 0.1% gentamicin (Life technologies) and freshly added 0.1 μM dexamethasone (Sigma-Aldrich), 0.2 mM indomethacin (Sigma-Aldrich), 0.01 mg/ml insulin (Sigma-Aldrich) and 0.5 mM 3 iso-butyl-1-methyl-xanthine (Sigma-Aldrich). The cells were kept for 21 days in a 5% CO_2_ environment at 37°C and the differentiation media were refreshed twice a week.

### Histological and immunohistochemical stainings

#### Pretreatment for staining

The seeded cells were fixed with 3% formaldehyde for 15 min and then washed with PBS before further treatment. The paraffin embedded explants were cut in 6 μm sections and those sections were deparaffinized by successively dipping them for 5 min in Histoclear (National Diagnostics, Atlanta, GA), 100% ethanol, 96% ethanol, 70% ethanol and finally cleared in distilled water before further staining.

#### Alizarin red S staining

Cells were stained with Alizarin red S (Sigma-Aldrich) for 2 min and washed with microfiltered and deionized water. Paraffin sections were additionally and briefly immersed in acetone, then in an acetone-xylene (1∶1) solution and cleared in xylene. All samples were then counterstained with Mayer's hematoxylin for 5 min and rinsed in distilled water. Stained cells that contained mineral deposits appeared orange-red under the microscope.

#### Oil red O staining

Paraffin sections were first briefly dipped in 60% 2-propanol before starting the staining with Oil red O (Merck, Whitehouse, NJ) for 20 min and then dipped again in 60% 2-propanol and finally washed with distilled water. The seeded cells were directly stained with Oil red O. All samples were then counterstained with Mayer's hematoxylin for 5 min and repeatedly washed with tap water. Stained lipid droplets appeared red under the microscope.

### Gene expression analysis

Real time RT-PCR was used to monitor the differentiation potential of the two cell populations in the three lineage-specific induction media. Eight horses were used for analysis of the gene expression. Every sample was tested in triplicate for each gene. We used the following primers (Table 1) to determine the gene expression of the seeded cells: Col1A1, TNMD and SCX were chosen as tenogenic markers; Col3A1, MMP1 and MMP3 were considered as matrix remodeling markers; Runx2 and Sp7 were selected as osteogenic differentiation markers; PPARG and Fabp4 were used as markers of adipogenic differentiation; and Oct-4, CD34, CD45, CD90 and CD105 were chosen as progenitor cell markers [23]–[25]. GAPDH, β-actin and B2M were selected as internal controls (housekeeping genes) based on existing literature on optimal housekeeping genes for RT-PCR in the equine species [26].

**Table 1:** Designed primers.

Primer	Coded Protein	Length (base pairs)	Primer sequence (5′ ->3′)
**Tenogenic markers**			
COLIA1 (XM_001499586.3)	Collagen type I	164	F:TGCCATCAAAGTCTTCTGCAA R:CGCCATACTCGAACTGGAATC
TNMD (NM_001081822.1)	Tenomodulin	142	F:AGAAGACCCGTCGCGCCAGA R:CGGCAGTAGCGGTTGCCTCG
SCX (NM_001105150.1)	Scleraxis	81	F:GCCGGTCACATCCCTCGCCA R:TCCTCCGACAGCGGGCTCAC
**Matrix remodeling markers**			
COL3A1 (XM_001917620.2)	Collagen type 3	150	F:TACTTCTCGCTCTGCTTCATCC R:GAACGGATCCTGAGTCACAGAC
MMP1 (NM_001081847.2)	Matrix metalloproteinase 1 (interstitial collagenase)	92	F:CGAAGGGAACCCTCGGTGGGA R:TGGCCTGGTCCACATCTGCTC
MMP3 (NM_001082495.2)	Matrix metalloproteinase 3(stromelysin 1, progelatinase)	139	F:TTTTGGCCATCTCTTCCTTCA R:TGTGGATGCCTCTTGGGTATC
**Progenitor cells markers**			
OCT4 (XM_001490108.4)	Octamer-binding transcription factor 4	149	F:AACCACACTCGTACCACGTC R:GCCCGAAAGAGAAAGCGAAC
CD34 (XM_001491596.2)	Cluster of differentiation 34	155	F:AGCACTATTCCCGCAAGACC R:TCCACCGTTCTCCGTGTAAC
CD45 (XM_005608047.1)	Cluster of differentiation 45 or PTPRC (Protein tyrosine phosphatase, receptor type, C)	206	F:ATTCCACGGGTGTTCAGCAA R:GGACCTTGGGCAGCAATGTA
CD105 (XM_003364144.2)	Cluster of differentiation 105	104	F:GCAGCACCTACTCCAACTGT R:CACCTTTTTCCGCTGTGGTG
CD90 (XM_001503225.2)	Cluster of differentiation 90	82	F:CAGAAGGTGACCAGCCTGA R:GTGTGGCGGTGGTATTCTCA
**Differentiation markers**			
RUNX2 (XM_001502519.3)	Runt-related transcription factor 2	81	F:CCCACGGCCCTCCCTGAACT R:TGTGCCTGCCTGGGGTCTGT
SP7 (XM_001494930.3)	Transcription factor osterix, transcript variant1	130	F:GATGGCGTCCTCCCTGCTTGA R:GCCTGCTTTGCCCAGTGTCGT
PPARG (XM_001492430.1)	Peroxisome prolifertor-activated receptor gamma-like, transcript variant 2 (LOC100051258)	185	F:AGGGGCCTTTACCTCTGCTGGT R:TGGGCCAAAATGGCATCTCCGT
FABP4 (XM_001490771.3)	Fatty acid-binding protein, adipocyte-like (LOC100057425)	195	F:ACACCAGAGGGTCAGACACCT R:GGTTTGGCCATGCCAGCCAC
**House-keeping genes**			
GAPDH (NM_001163856.1)	Glyceraldehyde-3-phosphate dehydrogenase	141	F:GTCAACGGATTTGGTTATTGGG R:TGCCATGGGTGAATCATATTGG
βACTIN (NM_001081838.1)	Beta-actin	107	F:CACCACACCTTCTACAAC R:ATCTGGGTCATCTTCTCG
B2M (NM_001082502.2)	Beta-2-microglobulin	271	F:GGCTACTCTCCCTGACTGG R:ACACGGCAACTATACTCATCC

#### RNA isolation and real time RT-PCR

Medium was removed from the wells and cells were suspended in RNA-Bee (Tel-test, Friendswood, TX). Cells' RNA was then isolated using RNeasy Micro Kit 50 (Qiagen, West Sussex, UK) with on-column DNAase digestion. Total RNA content was determined spectrophotometrically using a NanoDrop (Thermo Fisher Scientific, Waltham, MA). The RNA obtained was further reverse-transcribed into cDNA with a RevertAid First Strand cDNA Synthesis Kit (Fermentas, Thermo Fisher Scientific). Finally, real time RT-PCR was performed using a real time PCR system thermal cycling block (StepOnePlus, Applied Biosystems, Carlsbad, CA) with Power Sybr green PCR master mix (Applied Biosystems) and standard software (StepOne version V2.1, Applied Biosystems).

#### RT-PCR data processing

Data were normalized to three housekeeping genes (GAPDH, β-actin and B2M) that were stably expressed across sample conditions (with the exception of osteogenic and adipogenic induction experiments, for which only GAPDH was stably expressed and alone used for normalization). Relative expression and fold-change expression were calculated according to the 2-ΔΔCT formula [27] using pooled triplicate samples (average of triplicate wells), and normalized to group median expression. Experimental groups with median CT values above 30 were considered to have low or negligible gene expression, and all presented data or derivative fold change calculations are indicated as such. Statistical analysis was performed using SPSS 11.5 software (SPSS Inc., Chicago, IL). Fold change values are presented as Box-Whisker plots, with the boxes representing the middle two quartiles (25–75) and the whiskers representing the highest and lowest data point values.

### Western Blotting

The cells from three different horses were lysed in sample buffer (0.1 g/ml SDS (Biosolve BV, Valkenswaard, NL), 1 M Tris-HCl (Biosolve BV), 50% glycerol (Sigma-Aldrich)). Equal amounts of proteins were analyzed by Western blotting using antibodies to collagen type I (Abcam, Cambridge, UK), scleraxis (Abcam), α-SMA ((mouse IgG2a, clone SM1) kind gift of Prof. Christine Chaponnier and Prof. Giulio Gabbiani, University of Geneva, Switzerland [28]) and vimentin (DAKO, Glostrup, Denmark). The band signal strengths in Western blots were further analyzed by optical densitometry and related to loading control vimentin. The experiments were run in triplicate.

### Myofibroblast differentiation

The potential of the two cell populations to differentiate toward a myofibroblastic phenotype was assessed by comparing relative expression of α-SMA in cells in culture with the presence or absence of transforming growth factor beta 1(TGF-β1 (2 ng/ml); R&D systems, Minneapolis, MN). TGF-β1 is known to elicit the expression of α-SMA, the most frequently used marker of myofibroblasts. Additionally, the differentiation potential of the two cell populations was also tested on different ligands. Peritenon cells as well as cells from the core of the tendon from three different horses were seeded on 100 kPa Polydimethylsiloxane (PDMS; ExCellness Biotech SA, Lausanne, Switzerland) coated with collagen (Sigma-Aldrich) or fibronectin (EMD Millipore, Billerica, MA). The differentiation potential toward a myofibroblastic phenotype was then determined for both cell populations using the Western blot techniques described above.

### Statistical analysis

Related Samples Wilcoxon Signed Rank tests were used to assess differences among conditions. Significance level was set at p<0.05.

## Results

**Figure 1:**
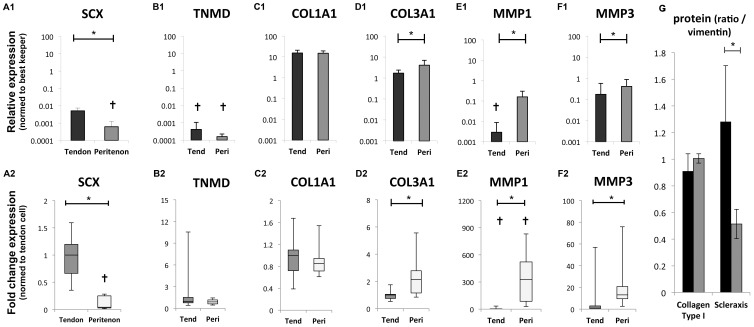
Gene expression levels of tenogenic markers, matrix remodeling markers, and western blots on tenogenic markers to confirm gene expression results. Gene expression of (A) SCX, (B) TNMD, (C) COL1A1, (D) COL3A1, (E) MMP1, and (F) MMP3 in tendon core cell population and peritenon cell population cultured in expansion medium. The cells were extracted from eight different horses. Using the 2^−ΔΔ*C*T^ method, the data are presented both in terms of “relative expression” normalized to three housekeeping genes, as well as the fold change in gene expression normalized relative to the tendon core expression. The respective p values are as follows:(A) SCX (24-fold; p = 0.01), (B) TNMD low expression and not significantly different, (C) no difference for COL1A1 (p = 0.48) (D) Col3A1 (2.1-fold; p = 0.02), (E) MMP1 (330-fold; p = 0.02), (F) MMP3 (13-fold; p = 0.02. (G) Western blots on tenogenic markers to confirm the gene expression results. The graphs display the relative band signal strength in Western blots analyzed by optical densitometry and related to loading control vimentin. Cells from the tendon core population demonstrated a higher level of scleraxis protein expression compared to cells from the peritenon when cultured in expansion medium for a week (p<0.05). The difference between the two cell populations for collagen protein expression was not significant.

Comparison of gene expression in both populations of cells for tenogenic markers (SCX, TNMD and COL1A1; fig. 1) showed that cells from the core of the tendon expressed significantly higher amounts of SCX (24-fold; p = 0.01), with statistically indistinguishable amounts of COL1A1 expression in the two cell populations (p = 0.48). Expression of TNMD was very low (CT values >34) in all samples and statistically indistinguishable. Western blot results for scleraxis and collagen type I confirmed the gene expression data for the corresponding genes.

Looking further at expression of genes involved in tendon extracellular matrix remodeling, cells from the peritenon expressed higher amounts compared to cells from the tendon core for COL3A1 (2.1-fold; p = 0.02), MMP1 (330-fold; p = 0.02; with minimal expression in tendon cells) and MMP3 (13-fold; p = 0.02).

**Figure 2:**
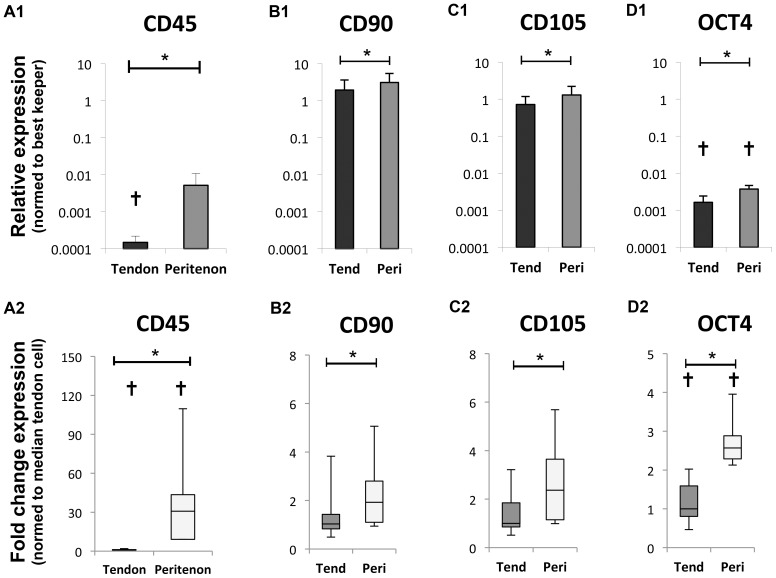
Gene expression levels of progenitor cell markers, indicates higher levels of progenitor cell markers in peritenon cell populations. (A) CD45 (31-fold; p = 0.03), (B) CD90 (1.9-fold; p = 0.02) (C) CD105 (2.4-fold; p = 0.02), and (D) Oct-4 (2.6-fold; p = 0.02).

Looking at progenitor cell markers (fig. 2), cells from the peritenon expressed significantly higher amounts of CD45 (31-fold; p = 0.03; with low expression in tendon core cells), CD90 (1.9-fold; p = 0.02), CD105 (2.4-fold; p = 0.02), and Oct-4 (2.6-fold; p = 0.02; with low expression in both cell types), compared to cells from the tendon core. CD34 was expressed in both populations in negligible amounts and with no significant difference between the two populations (data not shown).

**Figure 3:**
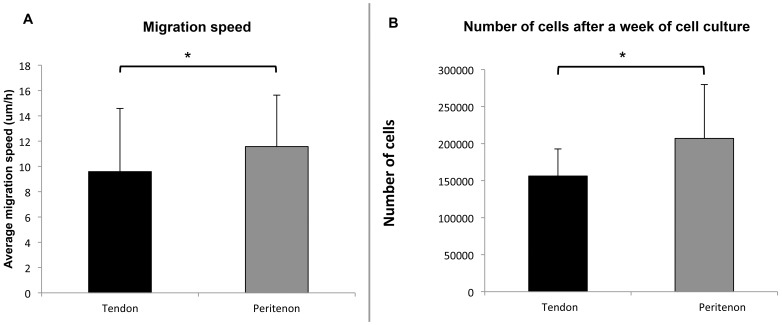
Migration and replication rate. (A) Average migration speed of the cell populations from the core of the tendon and from the peritenon, obtained with a scratch assay monitored over 8 h. The peritenon cell population was 1.2 times faster than the tendon core population (p  = 0.04). The cells were from twelve different horses. (B) Replication rate: Starting with equivalent numbers of cells, we calculated the number of cells after 7 days in culture in expansion medium. There were 1.33 times more cells in the flasks containing cells isolated from the peritenon compared to the flasks containing cells isolated from the tendon core (p = 0.026). The cells were from four different horses.

To test the hypothesis that peritenon cell populations demonstrate enhanced propensity for migration, confluent populations of cells from the tendon core and peritenon were monitored using a scratch assay. Peritenon cells showed significantly faster wound closure (1.2-fold; p = 0.04) compared to cells from the tendon core (fig. 3). Additionally, we evaluated the replication rate of the two cell populations. After 7 days, we counted 33% more cells in the peritenon population compared to the cell population from the core of the tendon (p = 0.03; fig. 3).

**Figure 4:**
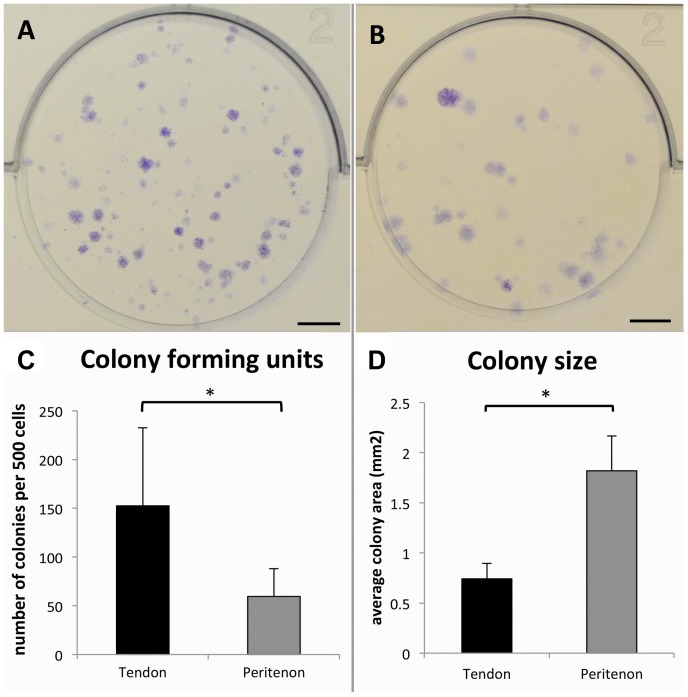
Clonogenicity of the two cell populations. Representative wells with colonies from the tendon core population (A) and from the peritenon population (B) were evaluated (n = 14; the cells were from seven different horses; scale bar  = 0.5 cm). The cells isolated from the tendon core demonstrated 2.6 times more colonies compared to the peritenon cell population (C; p = 0.02). The colonies were 2.5 times larger in the peritenon cell population than in the cells isolated from the tendon core (D; p = 0.018).

Clonogenicity, multipotency and self-renewal are widely used criteria to define stem cells. To characterize clonogenicity, cells from the tendon core and from the peritenon were seeded at a low density. During the first 2 days, the cells of both populations adhered but stayed quiescent. A small percentage of both populations of cells formed colonies on day 9 (fig. 4A,B). Cells from the tendon core formed 2.6 times more colonies than cells from the peritenon (p = 0.02; fig. 4C). Based on the assumption that each observable colony originates from a single progenitor cell, these data indicate a significantly higher proportion of progenitor cells in the tendon core population than in the cell population from the peritenon. The size of the colonies observed varied within one population, however the colonies formed by cells from the peritenon were overall larger (1.82 mm^2^±0.4 mm^2^ and 0.74 mm^2^±0.2 mm^2^ for peritenon and core cell populations, respectively (2.5-fold increase; p = 0.02; fig. 4D)) indicating higher proliferative capacity of peritenon progenitor cells and their progeny, despite formation of relatively lower numbers of colonies.

**Figure 5:**
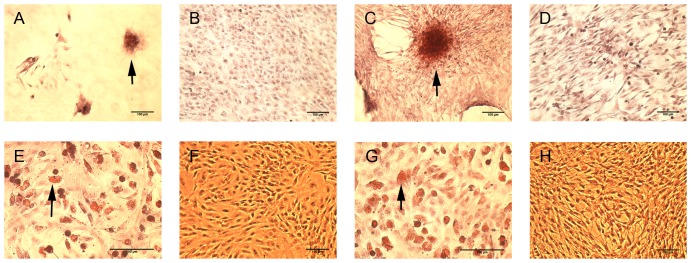
Potential for osteogenic and adipogenic differntiation of cells from the tendon and peritenon after 21 day culture in induction medium. Osteogenic (Alizarin Red; A–D) and adipogenic (Oil red O; E–H) differentiation of cells from the core of the tendon and from the tendon core (A,B; E,F) and from the peritenon (C,D; G,H). Larger calcification nodules (black arrows) were observed in the peritenon cell population (C) compared to the cells isolated from the tendon core (A) in osteogenic medium. Controls lacked nodule formation (B,D). Lipid vacuoles (black arrows) inside the cells of both populations when cultured in adipogenic medium (E,G), but not in the control condition with expansion medium (F,H). Scale bar  = 100 μm.

**Figure 6:**
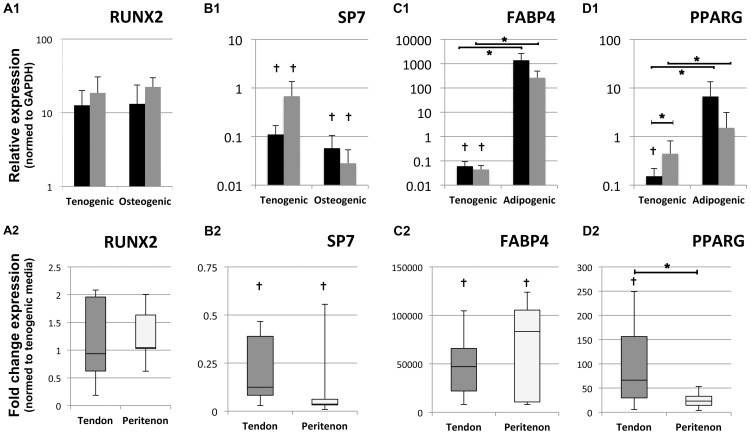
Gene expression of markers for osteogenic and adipogenic induction. Fold changes were calculated by dividing the relative expression of the gene of interest in the differentiation medium by the relative expression in the control expansion medium. No significant differences were found for osteogenic markers, either between induction and control media or between cell types (A,B). With respect to the adipogenic markers (C,D), both markers were substantially upregulated in induction medium. No significant difference was found between cell populations for FABP4 (C), while PPARG (D) was significantly upregulated (3.8-fold) in the cells isolated from the tendon core compared to the cell population from the peritenon (p<0.03); The cells were extracted from eight horses).

As tendons are derived from mesoderm origins, which also give rise to bone and fat, the adipogenic and osteogenic potential of the cells was tested using specific media and cell densities. When cultured in osteogenic medium, both populations showed an ability to differentiate towards bone (fig. 5A–D). Using Alizarin red staining, larger calcium plaques were observed for the population of peritenon cells (fig. 5C). The control conditions were devoid of observable calcium deposits (fig. 5B,D). Like the cell population from the tendon core, the peritenon cell population showed a heterogeneous differentiation potential toward adipogenic and osteogenic phenotypes. When cultured in adipogenic medium, cells from both populations exhibited numerous lipid droplets at day 21 (fig. 5E–H), indicating their ability to differentiate toward an adipogenic phenotype. Correspondingly, cell morphology drastically changed from a fibroblast-like spindle shape to a round shape. Oil red O staining revealed no clear differences between the two cell populations in adipogenic differentiation medium. No lipid droplets were found in the cells from either of the populations grown in a control medium lacking adipogenic supplements (fig. 5F,H). At the level of gene expression, no significant difference was observed for either the osteogenic markers Runx2 or SP7 in either osteogenic medium or control medium (fig. 6A,B). The expression of the adipogenic markers FABP4 and PPARG was significantly increased for both populations in adipogenic medium compared to the control medium. Median FABP4 expression increased by 47'000-fold and 83'400-fold for cell populations from the tendon core and from the peritenon, respectively, reflecting negligible expression of fat markers before activation in adipogenic medium (p<0.05). Median PPARG expression showed a 66-fold increase for the cell population from the tendon core and a 23-fold increase for the cell population from the peritenon (p<0.05). The cells from the core of the tendon had a significant increase (3.8-fold) in expression for PPARG in the adipogenic condition compared to the cells from the peritenon (p = 0.03), although baseline expression of PPARG was higher in peritenon cells. FABP4 expression showed no significant differences between the two cell populations in adipogenic medium.

**Figure 7:**
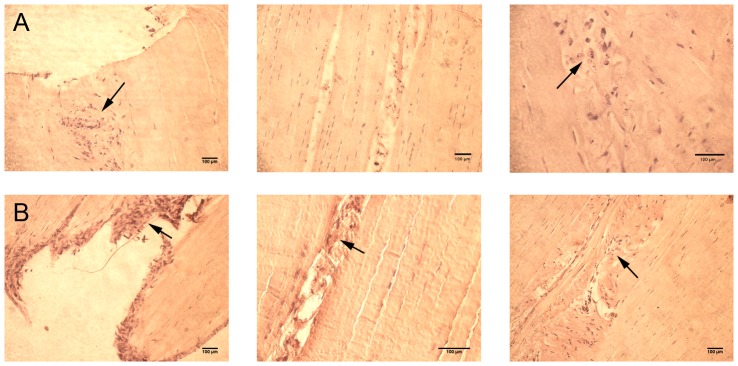
Cells at the margins of a tendon explant, but not those inside the more densely packed, well-organized intact matrix, show propensity for differentiation to non-tendinous phenotypes. Arrows indicate cells that are stained for markers of differentiation in adipogenic medium (Oil red O staining; row A) and osteogenic induction medium (Alizarin red S staining (row B). Images correspond to the edge of the sample, the middle of the explant section and in the endotenon (columns from left to right). Differentiated cells (black arrows) appear in the endotenon and peritenon, but not in the tendon core. Explants were analyzed from six different horses.

Additional explant experiments were undertaken to investigate how cells in their intact matrix would react to adipogenic and osteogenic differentiation media. Here, horse SDFT explants with their intact peritenon sheath were cultured for 21 days. Under adipogenic conditions, Oil red O staining revealed no changes for cells inside the tendon core that remained aligned along the collagen fibers (fig. 7A) In contrast, cells at the edge of the explants as well as those in the endotenon and peritenon showed a response to the differentiation medium. Numerous cells showed an enlarged and rounded shape. Similarly the cells in the dense, intact matrix of the tendon core were not affected by the osteogenic differentiation medium, whereas cells in the endotenon and peritenon showed a positive staining for Alizarin red (fig. 7B). The cell density in these regions also appeared to have dramatically increased.

**Figure 8:**
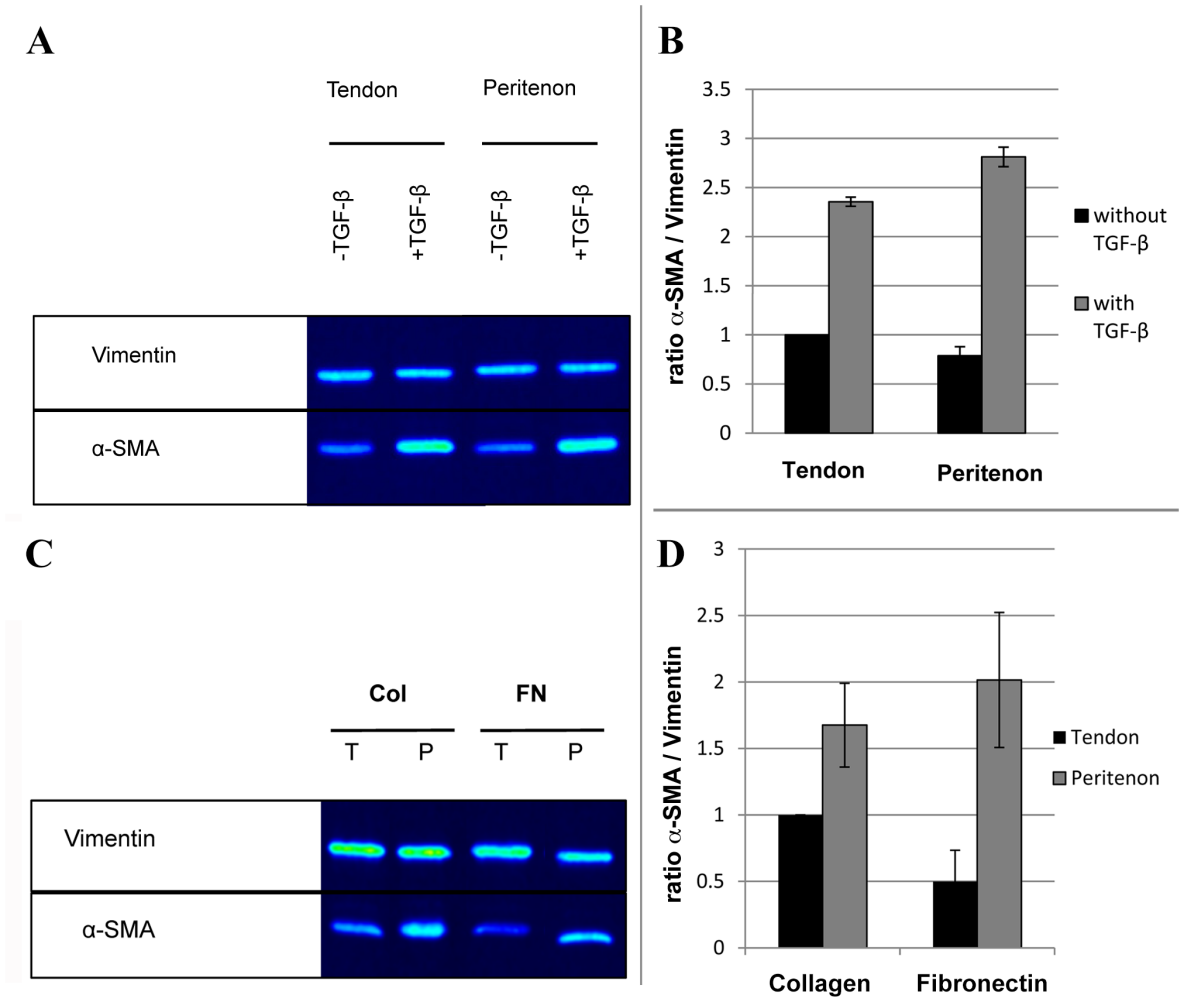
Comparison of the propensity to differentiate into myofibroblasts of the cell populations from the peritenon and from the core of the tendon and the effects of ligands on the differentiation propensity of cells isolated from the peritenon and cells isolated from the tendon core. (A–C) Expression of myofibroblast marker α-SMA was assessed by Western blotting together with loading control vimentin. (B–D) Band signal strength in Western blots was analyzed by optical densitometry and related to loading control vimentin. (A–B)Following addition of TGF-β1, the level of α-SMA was 2.4 times increased in the cell population from the tendon core and 3.6 times increased in the cell population from the peritenon. The cell population from the peritenon showed a 1.2-fold increased expression of α-SMA compared to the cell population from the tendon core when cultured with TGF-β1. (C–D) The cell population from the peritenon showed a 1.7-fold increase of α-SMA expression on 100 kPa PDMS dishes coated with collagen compared to the cell population of the tendon core and a 4-fold increase of α-SMA expression on same dishes coated with fibronectin (for both experiments, the cells were extracted from three different horses).

The propensity for myofibroblast differentiation was investigated by monitoring the level of α-SMA, a protein involved in the heightened contractility that characterizes myofibroblast behavior. In both cell populations, the presence of myofibroblasts in the original population could be demonstrated *in vitro*, even prior to stimulation with TGF-β1 (fig. 8A). More α-SMA levels were observed in the original population of cells isolated from the tendon core compared to the original peritenon cell population. As expected, the level of α-SMA increased for both populations of cells after the addition of TGF-β1 (α-SMA expression was increased 2.4 times for the cell population of the tendon core and 3.6 times for the cell population of the peritenon; fig. 8B). However, the level of α-SMA was 1.2 times higher in the peritenon cell population, indicating a higher differentiation potential towards myofibroblasts when compared to the cell population from the core (fig. 8B).

When seeded in presence of TGF-β1 on PDMS substrates (100 kPa) coated with collagen type I, the cell population from the peritenon showed a higher level of α-SMA (1.7 fold) compared to the cell population from the tendon core (fig. 8C). This non-significant tendency was augmented when the cells were seeded on PDMS coated with fibronectin, an extracellular matrix protein typical within injured tendons [29]. The cell population from the peritenon showed a 4-fold increase of α-SMA expression on fibronectin compared to the cell population from the tendon core (fig. 8D).

## Discussion

We have evaluated two cell populations of different origin for proxy measures of their potential healing capacity as well as for their potential involvement in pathogenesis. One population was directly derived from the core of the tendon (related to intrinsic healing) and the other came from the surrounding tissues of the tendon, the peritenon (extrinsic healing).

Tenogenic gene expression analysis showed distinctive profiles for each of the two cell populations. The cell population from the tendon core demonstrated a significantly higher expression of the gene coding for scleraxis when compared to the cell population coming from peritenon, similar to observations that have been made in murine Achilles tendon [11]. We observed no differential expression of the gene coding for tenomodulin, with TNMD expression being very low in both populations after their extraction.

Analysis of matrix remodeling-related gene expression, and measurements of replication and migration rates all indicated a faster reactive potential for cells from the peritenon compared with cells from the tendon core. These results are in accordance with functional *in vivo* studies in which cells from the epitenon have been observed to proliferate and migrate towards and into the wound [30]–[33]. The finding of similar levels of expression for Col1A1 (also confirmed at protein level) but increased levels for Col3A1 and MMP1 and MMP3 in the peritenon cell population compared to the tendon core population is consistent with the concept of peritenon-mediated extrinsic repair of tendon collagen structures, for instance after tendon suturing [30]. We were able to confirm these characteristics with controlled parameters *in vitro*. Taken together, these results suggest a dual healing process with a possible predominance of extrinsic healing.

While stem cells are heavily involved in homeostasis, growth and repair of many tissues [34], they may also contribute to some forms of tendinopathy by creating ectopic tissue inside the tendon [35].Our RT-PCR analyses demonstrated that the cells from the peritenon had significantly higher expressions of the following genes: CD45, CD90, CD105, and Oct-4 compared to the cells from the tendon core. None of the populations expressed CD34 and only cells from the peritenon expressed CD45. Gene expression of CD90 and CD105 are accepted markers for human and equine mesenchymal stem cells (MSC) [23]–[25]. Oct-4 has been used as an MSC marker for cells originating from adipose tissue, bone marrow and umbilical cord [25], [36]–[38] and CD45 and CD34 are used as markers to differentiate MSC from hematopoietic stem cells. Taken together, these results suggest a higher proportion of MSC and the presence of hematopoietic stem cells in the cells population from the peritenon compared to the population originating from the tendon core. Our assays of clonogenicity indicated a slightly ambiguous result, with fewer but substantially larger colonies formed in cell populations from the peritenon compared to the cell populations from the tendon core. While these data suggest a potentially higher proliferative rate of stem/progenitor cells in the peritenon cell population, additional investigations (e.g. fluorescence activated cell sorting (FACS) analysis of surface markers) to more accurately quantify progenitor cells are warranted. Our results also indicated increased formation of calcium deposits after differentiation in osteogenic media for peritenon cell populations compared to populations extracted from the tendon core. However this qualitative difference in calcium deposition was incongruous with statistically equivalent gene expression of osteogenic markers. Similarly, potential for adipogenic differentiation appeared roughly equivalent for both cell populations. Collectively these data clearly suggest the existence of stem/progenitor cells in both populations, but with greater migration rates, proliferative capacity, and a possibly higher potential for calcium formation in cell populations extracted from the peritenon. These findings are consistent with those of Mienaltowski et al., who reported that the number of colonies formed by progenitor cells from the tendon core was higher than from the peritenon in mouse Achilles tendon [11]. As in the present study, these authors also did not observe any clear difference regarding adipogenic differentiation potential between the two cell populations. One interesting distinction between our results and those of Mienaltowski et al. is that they did not observe any calcium deposits in the cell population from the peritenon whereas we observed clear calcification in this cell population

In contrast to one other report [39], we did not detect adipogenic markers in cells cultured in control medium. One plausible explanation for this discrepancy could be that equine tendon cells display a different behavior from those of rodents, paralleling similar differences in adipogenic and osteogenic potential of tendon/stem progenitor cells between mice and humans [40]. Species-related specificity in differentiation may also explain why, unlike in human tendons, clinical observations of lipid accumulation in diseased or injured equine tendons have not yet been reported [41].

We observed that cells at the margins of a tendon explant, but not those inside the more densely packed, well-organized intact matrix, show propensity for differentiation to non-tendinous phenotypes. This suggests that a well-structured matrix may play a key role in regulating the fate of stem/progenitor cells, with possible implications for adipogenesis in tendinopathy, and ectopic calcifications. Previous *in vitro* studies using cross-linked collagen matrices and gels have also shown a clear influence of matrix compliance in directing the stem cell lineage specifications [42], [43]. To our knowledge, our study is the first to show the influence of the matrix in tendons *ex vivo*.

Myofibroblasts are key players in the classic connective tissue wound healing paradigm but are also associated with excessive scar formation [44], [45]. Little is known of the role of myofibroblasts in healing tendons [46]. Our results reveal the presence of α-SMA in native cell populations of both tissue compartments *in vitro*, with higher levels observed in cells from the tendon core. In theory, these observations might be explained in part by the presence of pericytes (contractile cells lining blood capillaries) within the sampled populations and not necessarily by a differentiation of fibroblastic cells, such as tenocytes, into myofibroblasts. However, tendons are poorly vascularized, as confirmed by De Mos et al. who found that 98.5±0.7% of the cells isolated from tendons were negative for CD34, an endothelial cell marker [15]. More importantly for the present investigation, α-SMA expression increased in both cell populations after the addition of TGF-β1, with the peritenon-derived cell population showing a greater potential to differentiate into myofibroblasts. Furthermore, our findings indicated increased α-SMA levels in cells seeded on fibronectin-coated substrates compared to collagen-coated ones, suggesting a high affinity for fibronectin, which is abundantly found in healing tendons [29]. These results indicate again that the cells from the peritenon are highly reactive cells. It is therefore very plausible that they are implicated in tendon healing processes.

Aside from careful anatomical extraction, we did not localize the origin of cells showing a clear tenogenic phenotype or stem/progenitor cell markers. Consequently, we were not able to evaluate the proportion of those cells in the population from the core of the tendon that had come from the endotenon. We were also not able to distinguish between individual cells with multilineage potential and more or less strongly committed cells. We did find that not all of the cells showed a clear differentiation towards other lineages (osteogenic, adipogenic and myofibroblastic), which indicates that there were different subpopulations within the larger populations of cells extracted from these two tissue sources. While surface marker analysis of the cells could provide additional clarification in future studies, FACS analysis of mature tendon cells and their progenitors is currently limited with established surface markers only now emerging [15]. Still, the functional data we provide give further evidence for other studies where stem/progenitor cells have been reported to originate from the tendon core [15], [39], [40], [47] and from the peritenon [11]. Our data also confirm the reportedly higher reactivity of stem/progenitor cells from the peritenon compared with those from the tendon core [11]. Beyond previous studies, our study is the first to indicate a higher differentiation potential towards a myofibroblastic phenotype for the cells from the peritenon in comparison with cells from the core of the tendon. This suggests that there may be a greater potential of cells from the peritenon to be involved in formation of scarring and adhesions during tendon healing. We also investigated for the first time the role of a native (explanted) 3D tendon matrix in influencing cell differentiation towards adipogenic or osteogenic lineage and we showed a clear protective effect of an intact matrix. More importantly, our results show that both the cells from the peritenon and the cells from the core of the tendon were able to express and produce large amounts of collagen type I, indicating a possible dual mechanism (intrinsic and extrinsic) for tendon healing.

Taken together, these findings highlight an unexpectedly large plasticity of both tendon and peritendinous cells – with potentially important implications for tissue repair mechanisms. These mechanisms are central to our understanding of the onset and development of tissue pathologies such as tendinopathy, and the eventual development of improved therapeutic strategies.
